# Assessment of women’s needs and wishes regarding interprofessional guidance on oral health in pregnancy – a qualitative study

**DOI:** 10.1186/s12884-024-06675-w

**Published:** 2024-07-11

**Authors:** Merle Ebinghaus, Caroline Johanna Agricola, Janne Schmittinger, Nataliya Makarova, Birgit-Christiane Zyriax

**Affiliations:** Midwifery Science - Health Care Research and Prevention, Institute for Health Services Research in Dermatology and Nursing (IVDP), University Medical Centre Hamburg-Eppendorf (UKE), Martinistraße 52, 20246 Hamburg, Germany

**Keywords:** Oral health, Needs assessment, Health education, Interprofessional collaboration, Prenatal care, Midwifery care, Dental care

## Abstract

**Background:**

Poor oral and dental health due to oral dysbiosis during pregnancy increases the risk for negative pregnancy outcomes. Communicating the importance of oral health is therefore essential in reducing the risk of adverse pregnancy outcomes. Professional guidance could substantially support women’s positive perception of their own competence. Information on oral health should be provided by healthcare professionals such as midwives, obstetricians and dentists. The aim of this study was to assess the needs, wishes and preferences of pregnant women in Germany, regarding interprofessional collaboration and guidance on oral health during pregnancy.

**Methods:**

Sources of information, preferences regarding information supply as well as the need for interprofessional collaboration of involved healthcare professions were investigated in six online focus groups with pregnant women. In addition, three expert interviews with a midwife, an obstetrician and a dentist were conducted. The focus groups and interviews were analysed using qualitative content analysis according to Kuckartz.

**Results:**

25 pregnant women participated in focus groups. Pregnant women in all trimesters, aged 23 to 38 years, were included. Many women did not receive any or received insufficient information on oral health during pregnancy and wished for more consistent and written information from all involved healthcare providers. The extent of oral health counselling women received, heavily relied on their personal initiative and many would have appreciated learning about the scientific connection between oral health and pregnancy outcomes. An overall uncertainty about the timing and safety of a dental visit during pregnancy was identified. Interviews with experts provided additional insights into the working conditions of the involved healthcare professionals in counselling and emphasised the need for improved training on oral health during pregnancy in their respective professional education as well as thematic billing options in relation to this topic.

**Conclusion:**

Guidance of women on oral health during pregnancy appears to be insufficient. Providing information adapted to the needs, wishes and preferences of women during pregnancy as well as the implementation of this topic in the education of involved healthcare professionals could contribute to an improved prenatal care for pregnant women and subsequently a reduced risk of negative pregnancy outcomes.

**Supplementary Information:**

The online version contains supplementary material available at 10.1186/s12884-024-06675-w.

## Background

The importance of oral and dental health during pregnancy has been described by multiple studies in recent years [1–5]. The unique physiological state of pregnancy is concurrent to changes in the immune system and differences in hormone levels, leading to swollen gums and altering the composition of the oral microbiome, making women more susceptible to oral diseases through local inflammation [1]. Periodontal diseases (PD) and microbial dysbiosis have been shown to trigger systemic inflammation leading to an increased risk of adverse pregnancy outcomes (APOs) such as preeclampsia, low birth weight (LBW), preterm birth (PTB) and miscarriages [1–3]. Oral health in pregnancy is influenced by factors beyond general oral hygiene, including nutrition and changes in eating behaviour, contributing to the development of caries and PD [6, 7] as well as the frequent contact with gastrointestinal acid due to maternity sickness, which can additionally lead to dental erosion [6]. This is complicated by the fact that pregnancy related nausea and emesis can make the brushing of teeth difficult and the visit to the dentist daunting, consequently affecting oral health [7]. German studies of recent years observed that many women do not appear to be aware of risks associated with poor oral health, and that individual dental care and frequency of dental visits are associated with educational level [8, 9]. Enhanced oral health literacy through behavioural and educational counselling can however improve women’s oral hygiene and even reduce gingival inflammation [10]. In Germany, prenatal care givers should provide medical advice to pregnant women on oral health as stipulated by the maternity guidelines, which should be checked off in their maternity log, a document owned by every pregnant woman in Germany [11]. Oral and dental health prevention is covered through Germany’s statutory health insurance for every insured citizen (> 99,9%) [12], allowing for a free, but not mandatory, oral health consultation by a dentist during pregnancy as well. Limited research has shown that knowledge in relation to oral health and APOs is somewhat prevalent in healthcare professionals, differs however in levels of awareness between midwives, obstetricians, dentists and general practitioners [13, 14]. In improving healthcare and pregnancy outcomes, a collaborative approach with multiple healthcare workers from different professional backgrounds, working together in providing comprehensive services and increasing the quality of care that women receive, has been shown to have a positive impact [15]. Especially pregnant women with limited health literacy might benefit from redundancy of educational counselling in multiple settings and the involved healthcare professionals working closely together [16]. For oral health, the main professions involved in guiding pregnant women are midwives, obstetricians as well as dentists. While most pregnancies in Germany are accompanied by obstetricians, women are also entitled to prenatal care by midwives and women can choose their model of care to be alternating or primarily by one of the professions [11]. The training of midwives recently underwent academisation in Germany, offering the opportunity to implement novel contents and introduce interprofessional collaboration with other medical professions. It has been highlighted by multiple studies that midwives play a significant role in encouraging positive health behaviour in pregnant women, attributed to their oftentimes close bond and trusting relationship [17]. The quality of interprofessional collaboration in Germany remains to be investigated however, and focusing on the impactful topic of guidance on oral health in pregnancy is a research gap yet to be closed. When examining this topic, considering the collaboration between all involved professions is only consequential and best determined when knowing the needs of those who are experiencing prenatal care. Identifying women’s sources for information and evaluating their wishes constitutes an important insight in implementing a clear benefit for pregnant women regarding their pregnancy outcomes. The combination of pregnant women’s apparent unawareness, healthcare providers’ possible lack of knowledge and the severity of associated APOs makes the education on the topic of oral and dental health all the more important. This study gathered the collective perspectives of women in different phases of pregnancy, using the qualitative research approach of focus groups, as well as the viewpoints of three involved healthcare experts through interviews with a midwife, an obstetrician and a dentist. The aim of the study was to assess the current state of care, to identify areas in need of improvement and synthesise ideas for directions of future research in implementing interprofessional and demand-oriented prenatal healthcare in terms of oral health.

## Methods

### Study design and participants

This study followed a qualitative approach, aiming at the exploration of individual needs, wishes and preferences through focus group discussions and interviews. The methodology is described in detail in our companion manuscript, Ebinghaus et al., 2024, which focuses on nutrition guidance during pregnancy [18]. The study was structured into two parts. Firstly, six semi-structured focus groups [19] with pregnant women in all trimesters were conducted. Additionally, a semi-structured one-on-one interview [19] was conducted at the request of one participant, who did not feel comfortable in a focus group setting. Secondly, three professional healthcare providers were interviewed in semi-structured one-on-one interviews, including a midwife, an obstetrician and a dentist. In preparation for both, written guidelines were developed for focus group discussions and expert interviews, respectively (Supplement S1). 25 pregnant women participated in six focus groups during the months of September and October of 2022, which were conducted via the online-platform Zoom. Three healthcare professionals were interviewed over the phone, two interviews took place in November of 2022 and one in January of 2023. Data was collected via focus groups until no new ideas emerged and saturation was apparent, meaning that recruitment of participants continued in parallel to the conduct of focus groups. Using the MAXQDA software, the transcripts were categorised into a code system and thematically analysed according to Kuckartz [20]. The study was approved by the Local Psychological Ethics Committee of the University Medical Centre Hamburg-Eppendorf (LPEK-0507) and registered at Open Science Framework (OSF) on 12.09.2022 (DOI: 10.17605/OSF.IO/YP7BR).

Women at any stage of pregnancy, who experienced their pregnancy within the German healthcare system, who were able to join a focus group online, speak German and to give informed consent, were included. Pregnant women who themselves worked as a midwife, obstetrician or dentist were excluded, to avoid bias in their judgement of the German healthcare system and other participants in the focus group feeling uncomfortable expressing their opinions on involved professions. Following the focus groups, one representative of each profession was recruited for expert interviews. The term “expert” in this context was defined as a healthcare professional with the appropriate professional education completed in Germany and at least ten years of experience working in their respective field within the German healthcare system.

### Data management and analysis

The management and analysis of the obtained data was performed using the 2022 version of MAXQDA. Prior to participating in the study, all participants signed a written informed consent form and were assigned a pseudonym, which was saved in combination with the participant’s contact information on a password-protected excel sheet. To obtain an understanding and general view of the collected data, it was analysed using a concept-driven combined with a data-driven approach, reflecting the systematic approach to qualitative content analysis for a deductive-inductive analysis by Kuckartz [20]. The applied framework method was described by Gale et al. [21] and entails the organisation of codes into categories. The resulting category system was then revised by multiple researchers experienced in qualitative research, ensuring a clear structure and comprehensibility. To gain scientific validity, a second researcher independently applied the developed category system for one focus group transcript. Aiming for agreement of coding and inter-coder consistency, existing disagreements were discussed between the researchers until consensus was reached. The final category system for the focus groups was subsequently applied to the interview transcripts, to allow comparison between the statements of the involved pregnant women and healthcare professionals. Translation from German to English was performed as closely to the original categories as possible, to ensure their meaning reflects the expressed views of the participants. Direct quotes from the focus groups and interviews presented in the results section were translated in a literal manner from German into English.

## Results

### Sample characteristics

Six focus groups and one interview were conducted with pregnant women, as well as three expert interviews with healthcare professionals. A total of *n* = 25 pregnant women participated in the study. Women were between the ages of 23 and 38, with a mean age of 30.9 (SD: 3.4). At the time of their respective focus group, *n* = 2 participants were in their first trimester of pregnancy, while *n* = 10 were in their second and *n* = 13 in their third trimester of pregnancy. The week of pregnancy ranged from 8 to 40 weeks. Six women had one child prior to their current pregnancy, the other 19 women were expecting their first child. All participants had a high school diploma (“Abitur”) and *n* = 13 completed college education. The places of residence at the time of the study were located in seven different federal states of Germany, most participants lived in Northern Germany (*n* = 16). The participating women had chosen three different models of care for their pregnancy, with *n* = 10 receiving prenatal care exclusively from their obstetrician and *n* = 11 choosing an alternating model between midwife and obstetrician. The midwifery-led model of care was chosen by *n* = 4 women. The second part of this study consisted of three expert interviews with healthcare providers. The interview participants completed their professional education in Germany and had multiple years of experience in their field. At the time of the interviews, the obstetrician and dentist worked in a joint practice, respectively. This is a common concept in the German health care system, where multiple doctors of the same specialty manage a practice together. The midwife was self-employed in prenatal care, independently from a practice, which is also common in pre- and postnatal midwifery care in Germany. Table 1 summarises the socio-demographics of the focus group participants, and Table 2 those of the interview participants.

**Table 1:** Socio-demographic characteristics of focus group participants (*n* = 25)

Participant number	Age (years)	Number of children	Model of care	Week of pregnancy
01–01	26	0	Obstetric	38
01–02	30	0	Obstetric	37
02 − 01	27	0	Obstetric	37
02–02	29	0	Alternating	39
03 − 01	31	0	Midwifery	20
03 − 02	34	1 (7 years old)	Midwifery	10
03–03	36	1 (1 year old)	Alternating	31
03–04	32	1 (1 year old)	Obstetric	31
03–05	33	0	Obstetric	20
03–06	30	0	Obstetric	34
04 − 01	35	0	Alternating	35
05 − 01	32	0	Midwifery	18
05 − 02	30	0	Alternating	29
05 − 03	26	0	Alternating	38
05 − 04	38	1 (8 years old)	Alternating	25
05–05	33	0	Obstetric	20
06 − 01	32	1 (3 years old)	Obstetric	28
06 − 02	31	0	Alternating	8
06 − 03	30	0	Alternating	23
06 − 04	23	0	Obstetric	37
06 − 05	34	0	Obstetric	40
07 − 01	34	0	Alternating	27
07 − 02	28	1 (2 years old)	Midwifery	37
07 − 03	28	0	Alternating	15
07 − 04	30	0	Alternating	28

**Table 2:** Socio-demographic characteristics of experts (*n* = 3)

Participant number	Profession	Employed since	Setting
08 − 01	Dentist	2005	Employed in joint practice
08 − 02	Obstetrician	1999	Self-employed in joint practice
08 − 03	Midwife	1999	Self-employed

### Category system

The development of the category system followed a deductive-inductive approach [20]. The deductive categories were determined using the focus group and interview guidelines to cover the main aspects the study participants were asked about. Those are the categories “source and type of information”, “oral and dental health related concerns in pregnancy”, “timing of consultation”, “prenatal oral health services in demand”, “interprofessional collaboration”, and “professional education of experts”. The subsequent inductive approach, working with the focus group and interview transcripts, resulted in the subcategories. One main category was included after inductive coding, which was the observed “lack of guidance”. The final category system is shown in Supplement S2.

## Guidance and education on oral and dental health during pregnancy

### Source and type of information

#### Perspectives of pregnant women

The focus group participants named healthcare providers as their main source of information on oral and dental health in pregnancy. Some women stated they were reminded by their obstetrician or in some cases their midwife to visit the dentist and got recommended a professional dental cleaning during pregnancy, usually without any further explanation as to why this is important. A more comprehensive consultation was sometimes provided by dentists, which was appreciated by the women and described as helpful. However, the participant’s experiences were very heterogeneous, with many receiving no information at all on the topic. Many women pointed out, had they not visited the dentist for their annual appointment, independently from their pregnancy or personal initiative, they would have had no knowledge of the relevance of this topic at all.

*“I already have an increased risk of periodontitis*,* so I regularly go for dental cleanings. But*, [the dentist] *was the one who told me that I should pay special attention now. Neither the midwife nor the obstetrician said anything about it.”* (36 years, 2nd child).

Women generally voiced the wish to be better or even at all informed about oral and dental health during pregnancy and would like more detailed information to be offered by both obstetrician and midwife. Those who had not received any further counselling by their dentist directly expressed their wish to be informed by that profession as well.

*“I think it’s important that the obstetrician mentions this as well. I mean*,* they can’t assume that pregnant women know about this. So*,* I think it’s important that it’s addressed with everyone*,* and ultimately*,* it would be good to be addressed by the midwife as well. And then I can also say that I will make an appointment with the dentist and get further advice.”* (31 years, 1st child).

Some women stated they had only received an education on oral health verbally by their healthcare providers, resulting in the loss of a lot of information due to the sheer amount and many women feeling nervous or excited.

*“And in this consultation*,* even if someone*,* which we haven’t heard so far*,* would have taken really extensive time for the education*,* it’s also a question of how much actually sticks*,* because sometimes you are simply*,* yes*,* maybe excited or whatever. Therefore*,* I think something in writing is helpful in any case.”* (26 years, 1st child).

There was an overall agreement about the importance of one-on-one conversations and verbally conveying the relevant information. However, the focus group participants consistently emphasised the importance of written information, to take home and to be able to read up on it again, providing them with more in detail information. This would help to avoid forgetting about important information or appointments, such as dental visits, but also leave women with a reliable source they can trust and would also give women the opportunity to think of questions they would then be able to clarify during their next appointment.

*“I would also find it nice to have something*,* like*,* that you get a booklet put together and receive it at the first appointment*,* and then maybe go over it again at the next appointment and see if there are any questions. But that you can also look at it again at home in peace.”* (28 years, 1st child).

One idea that emerged throughout focus group discussions was the benefit of a new information sheet women would receive by their midwife or obstetrician in each trimester, summarising important aspects and things to consider during this particular time. Positively perceived by those who received it, was the so-called children’s dental passport, reminding them about dentist appointments during pregnancy and their future child’s dental health. This led other women to express their wish to have received this by their midwife or obstetrician as well.

*“I received a brochure at my first appointment with the obstetrician where I also got a little booklet on children’s teeth*,* where you can write down when the child gets its first tooth and other such milestones. I thought it was quite sweet and useful to have a reminder like that.”* (34 years, 1st child).

Additionally, a list of internet links, where women could obtain information from reliable sources was appreciated by focus group participants. One woman proposed a link to a video, for example by the German Federal Ministry of Health, targeting every population group in Germany, to also cater to any language barriers or difficulties to understand complex medical topics.

*“And that’s why I think something like a video from the Ministry of Family Affairs or Health or something approved*,* even on certain social media platforms*,* or anything else*,* should be available to provide prenatal care for all groups in Germany. Perhaps also for people with a migration background.”* (30 years, 1st child).

#### Perspectives of experts

The need for written information was reflected by the interviews with midwife, obstetrician and dentist, stating written material would facilitate the education on oral health. Just as the focus group participants they recognised the amount of information that pregnant women are confronted with as overwhelming, however emphasised the importance of them.

*“So these flyers really make it easier*,* and things to take with you. Because there is a lot of information at the first appointment*,* but also important. So we usually say*,* this is a lot of information*,* that’s why you get all these flyers and let it sink in first.”* (Obstetrician).

They appreciated flyers predesigned by German Federal Centre for Health Education (BZGA) or the German Nutrition Society (DGE), providing pregnant women with reliable sources and preventing them from extensively researching online. Both the obstetrician and dentist referred to the children’s dental passport they like to hand out.

*“Well*,* sometimes what facilitates it is the children’s dental passport that they can take home and look at more carefully*,* and if they have any questions or doubts*,* they can come back and ask them at the next appointment.”* (Dentist).

### Oral and dental health related concerns in pregnancy

#### Perspectives of pregnant women

The oral and dental health related topics that women were concerned with throughout their pregnancy included the worry about tooth decay, sensitivity of their gums, general oral hygiene and the dental health of their future child. Focus group participants also discussed pregnancy-related issues that make dental cleaning difficult and their need to be better informed on a tooth-friendly diet as well as about the scientific background of changing oral health in pregnancy. A pressing issue for many women seemed to be the sensitivity of their gums, describing bleeding of the gums, gingivitis and problems with their wisdom teeth. They reported to have received some albeit very limited education on this, in most cases only upon inquiring about it themselves.

*“I was also irregular with my dental appointments and then I said*,* I keep having bleeding gums*,* what’s going on? And my wisdom tooth started acting up all of a sudden. And then they said it’s completely normal*,* but no one had told me that before.”* (33 years, 1st child).

One woman reported having received a list of products she should buy and another would have liked this as well, after hearing about it.

*“I find it really interesting what you said about the different products*,* because for example with mouthwash*,* there is often alcohol in it*,* so I think a list like that would be really helpful.”* (26 years, 1st child).

Those who had not received any counselling on oral and dental hygiene in general voiced the wish to be better informed.

*“But otherwise*,* regarding dental hygiene*,* there wasn’t much information provided. Now that I hear this from others*,* I think it would have been useful to be advised on that.”* (30 years, 1st child).

A few focus group participants mentioned how they try to implement a tooth-friendly nutrition, avoiding lemonades or coffee and drinking a lot of water. Some women expressed the wish to be informed by their midwife or obstetrician on vitamins that might benefit dental health. Others would have liked to be more extensively informed by their dentist on dietary supplements, what would be beneficial for gums and teeth, and how to avoid caries.

*“I would have also appreciated it if my obstetrician had talked about it right away*,* because the topic of nutrition and teeth and vitamins are kind of interconnected. I would have liked to know a little more from the dentist about what can be done about it*,* besides a professional dental cleaning. Like*,* which vitamins can you take to support from that side? What would be good for the gums? Or what is generally important for your teeth? Because apparently you are also quite prone to cavities.”* (26 years, 1st child).

An overall wish by many women was to have the scientific backgrounds of oral health in pregnancy explained to them, to better understand, why a dental visit is recommended, how the two topics are related and what would happen if they did not practice oral care. Women were also interested to learn why their oral hygiene behaviour is connected to the development of caries in their future child.

*“I actually would have liked to know why. I still haven’t quite figured out what the background is. Why teeth are so important. What can happen in the worst case if I don’t pay attention to them. I just think I would have liked to know more.”* (35 years, 1st child).

Some women experienced difficulties in maintaining their oral and dental health due to maternity sickness, describing the brushing of teeth and using dental floss as a challenge, due to a heightened gag reflex.

*“And then my dentist said that it’s especially important to use dental floss and such. And I really thought*,* phew*,* brushing my teeth was already a big challenge.”* (36 years, 2nd child).

Maternity sickness also led to the fear of being sick during the dentist appointment on the dental chair. The dentist’s soothing reaction and offering to not do an examination as long as there are no apparent dental problems was appreciated.

*“I made an appointment*,* went there and I was so scared. I had really sweaty hands because I was afraid that I might throw up at the dentist’s office. I don’t really have a fear of dentists*,* but I was afraid that I would vomit due to my gag reflex. And lying on that chair was my absolute nightmare.”* (34 years, 1st child).

The uncomfortableness of a dentist appointment during pregnancy was an issue for many women, especially in early pregnancy, due to maternity sickness and in the third trimester due to inferior vena cava syndrome (IVCS).

#### Perspectives of experts

The interviews with midwife, obstetrician and dentist in some parts mirrored the pregnant women’s experiences and the topics they were concerned with regarding oral health in pregnancy. The dentist saw a special need for consultation in pregnancy regarding the sensitivity of gums, as many women experience this in their pregnancy. She considered this the most important aspect in her consultation and treatment and recommends a professional dental cleaning early in pregnancy. The midwife and obstetrician also named the bleeding of gums as the most frequent issue for pregnant women regarding their oral health.

*“It is also quite common for patients to come back in between appointments because gum bleeding has become so severe and they have sensitivities there*,* and there is a need for counselling and treatment. So I would say that this is probably the most important pillar that is also the most in demand.”* (Dentist).

The dentist also deemed consultation on periodontitis and its association with preterm birth as very important, which is something the obstetrician said she addresses in the first appointments with pregnant women as well.

*“Basically*,* the bacteria in the oral cavity*,* that you just say*,* the risk of preterm birth*,* the topic of periodontitis*,* that it is really important that the oral cavity is healthy*,* that plaque is removed accordingly*,* and such things.”* (Dentist).

A tooth-friendly nutrition was discussed only by the dentist, stating that in her experience, the regular consumption of sugary beverages is the biggest problem and many women do not know about the importance of nutrition for dental health.

*“But what really wakes everyone up*,* because what I have noticed*,* is not necessarily the nutrition itself. It is often drinking habits. And that is of course much more catastrophic for the teeth*,* every half hour*,* hour*,* sugar and acid are constantly coming into the mouth and that is catastrophic.”* (Dentist).

As also discussed by the focus group participants, a major pregnancy-related issue making oral hygiene more difficult is maternity sickness and a heightened gag reflex. Both the midwife and the dentist reported women’s worry to not be able to sufficiently practice oral hygiene and their need for information regarding an adjusted dental care routine.

*“That is a big topic*,* actually. Of course*,* I’ve tried to list or identify alternatives*,* such as the timing of brushing teeth*,* or rinsing with a plaque-dissolving solution for the mouthfeel first. And even if you can’t put the entire toothbrush in your mouth*,* you can already achieve a lot with dental floss or interdental brushes.* (Midwife)

According to the dentist’s experience, these pregnancy-related issues can in some cases make the treatment impossible and not all preferable procedures can be carried out. This is aggravated by IVCS, complicating treatment late in pregnancy.

*“Based on experience*,* factors such as gag reflex and nausea make treatment completely impossible. There are some patients who feel unwell at the beginning*,* and then treatment becomes unthinkable.”* (Dentist).

Adding to women’s uncertainty about dental treatments in pregnancy is the potential use of anaesthetics as pointed out by both the dentist and the midwife. Pregnant women are hesitant and want to limit treatments to the absolute necessary, according to the dentist. The midwife reported, she oftentimes witnessed the uncertainty of women regarding a dental visit due to inconclusive information in literature and on the internet.

*“Pregnant women also find it difficult to visit the dentist because they keep hearing from literature and the internet that they are not allowed to lie on their backs on the dental chair and that they should not undergo dental treatment during pregnancy because they are not allowed to receive local anaesthesia. And this is actually an insecurity that I have frequently observed.”* (Midwife).

### Timing of consultation

#### Perspectives of pregnant women

For many women, the time they received the advice to pay a visit to the dentist was their first appointment with the obstetrician early in their pregnancy. However, this advice was oftentimes the extent of oral health information during pregnancy by any healthcare provider. Most women considered the timing of consultation on oral and dental health to be ideal early on in their pregnancy. Even though many women felt overwhelmed by the amount of information at the beginning of their pregnancy, they emphasised the need to be informed about oral health early in their pregnancy.

*“In general*,* all the information was overwhelming to me during the beginning of the pregnancy. But still*,* I think it makes sense to receive this information at the beginning.”* (27 years, 1st child).

This was especially important regarding the reminder to make a dentist appointment by their obstetrician or midwife, giving women the possibility to schedule a dentist appointment in a timely manner. Some women pointed out, it gives them peace of mind to be able to check this off their list and knowing they will receive the relevant information later on.

*“Especially with dental hygiene*,* it’s important right at the beginning so that you have a bit of lead time with appointments. It’s good to just tick that off the list and know that you will definitely get the information you need in the coming weeks. You don’t have to worry until then*,* or look back and say*,* oh man*,* I should have done that three months ago. So I would have preferred a really thorough consultation right at the beginning.”* (30 years, 1st child).

Women also saw the need to be reminded multiple times by their prenatal care providers to make a dentist appointment, on the one hand to avoid forgetting about it and on the other hand to lay additional emphasis on the importance of the topic.

*“Because I also forget to make regular dental appointments. And then they only have a free slot in three months. In hindsight*,* I think they could have mentioned it.”* (30 years, 1st child).

The timing of a dental visit left many women confused, as some dentists refused to treat women outside the second trimester, failed however to explain their reasoning behind this.

*“*[The dentist] *said*,* I also don’t need to come a second time and that it’s definitely not done in the third trimester. It’s way too risky*,* you don’t do it. I got curious again and actually wanted to know why. But he had to move on already.”* (32 years, 1st child).

Therefore, many study participants wished for clearer instructions and reasoning on the ideal timing in pregnancy to visit the dentist and to be given the opportunity to schedule an appointment for their second trimester in a timely manner.

#### Perspectives of experts

In their respective interviews, the midwife, obstetrician and dentist all briefly touched upon the topic of timing of the consultation. The obstetrician described a brief consultation during the first prenatal visit with a pregnant woman and that she hands out the dental passport with a reminder to visit the dentist. This is also something the dentist gives pregnant women, mentioning that she is, especially in the beginning, reliant on the women disclosing their pregnancy to her. Interestingly, the midwife somewhat mirrored the experiences many women described during the focus group discussions, stating that oral health is a topic she has neglected during prenatal visits and has only consulted on when women specifically addressed any relating issues during these visits, such as nausea induced by tooth brushing or bleeding gums. Similarly, the dentist described her experience that many pregnant women seek education when they have issues with sensitive gums in the first or second trimester of pregnancy.

*“And at that point*,* it’s a consultation. But only if the woman describes a problem to me. If she then says*,* yeah*,* I’ll manage somehow*,* I haven’t pursued it any further. So*,* I haven’t really included it as a consultation topic*,* but more as something*,* only if it comes up.”* (Midwife).

Regarding the topic of pregnancy-related issues affecting the dental visit, the dentist very similarly to the focus group discussions, reflected on the uncertainty regarding the right timing of a dentist appointment during pregnancy. She explained this by the dividedness among dentists in general, with some refusing to treat pregnant women altogether, deeming it too risky. However, she stated treatment is possible throughout the entire pregnancy, while many dentists agree on the second trimester as the most convenient time, due to pregnancy-related issues of maternity sickness and IVCS being most apparent in the first and third trimester, respectively.

*“I also know of many colleagues who actually refuse to treat pregnant women. It has happened to me in other practices where I worked*,* where they really said*,* no*,* I don’t treat pregnant women*,* it’s too risky for me. If something happens to the child afterwards*,* I don’t want to be responsible.”* (Dentist).

### Prenatal oral health services in demand

#### Perspectives of pregnant women

Regarding medical services in oral healthcare, the topic of professional dental cleaning was addressed as well as the overall wish to be proactively offered more extensive guidance and information by healthcare professionals. Many focus group participants criticised that professional dental cleaning is cost-intensive and therefore not feasible for every pregnant woman. The women agreed on the need for professional dental cleaning to be covered by their health insurance for pregnant women, pointing out the fact that it is recommended during pregnancy while most insurance companies do not offer cost coverage.

*“But I have to say*,* that is of course again a cost factor. And if the health insurance company says*,* it is proven that pregnant women have a higher risk or are more susceptible to this and that. That maybe within these ten months of pregnancy a professional dental cleaning for pregnant women is free*,* or something. I believe that would make it much more attractive to make use of it.”* (32 years, 2nd child).

Regarding services that include the offer to consult and provide information, many women did not feel adequately informed on the topic of oral and dental health during pregnancy by their professional healthcare providers. The focus group participants voiced the wish to be asked about their general oral health status and potential problems that arose during pregnancy by their midwife and obstetrician.

*“I would have wished for more in that regard. Maybe even from the midwives*,* if there’s already so much talk about nutrition and such*,* to bring* [oral health] *up as well. If it’s already in the maternity log*,* as we’re talking about now*,* to make it a topic of conversation*,* like*,* hey*,* do you have any problems with your teeth or not? And the background behind why that’s actually important.”* (35 years, 1st child).

Many women felt like the discussion point of oral health was checked off in their maternity log by a prenatal care provider but not actually adequately executed. They see the responsibility with their midwife or obstetrician to only check off this item when it is accompanied by a complete and comprehensive consultation.

*“So*,* basically*,* it should be mandatory for healthcare professionals. That means if they advise a woman on this*,* they should do it comprehensively.”* (28 years, 2nd child).

#### Perspectives of experts

Regarding cost-coverage by health insurance, the dentist pointed out that some insurance companies reimburse the expenses of professional dental cleaning, regardless of pregnancy, at least in parts. She determined this as being sufficient, while also noting that she believes many women do not know about this.

*“However*,* it is also the case that nowadays prophylaxis*,* periodontal treatment*,* is well supported by health insurance. Many people do not know this*,* but with most statutory insurances*,* you can submit the bill and get a portion reimbursed. Therefore*,* I would say that the important things are actually covered.”* (Dentist).

The obstetrician saw potential in offering pregnant women a budget that they can use for different medical services, as already offered by some health insurance companies, which could include a professional dental cleaning. She proposed that financial reliefs for pregnant women would improve the implementation of oral healthcare in pregnancy.

*“Some health insurance companies have a budget specifically for pregnant women*,* where they can use up to 500 euros for medical expenses. Things like that are very helpful. This could actually help ease the financial burden on pregnant women.”* (Obstetrician).

### Exchange and cooperation between healthcare professionals

#### Perspectives of pregnant women

Many pregnant women would appreciate a cooperation between midwives and obstetricians, consulting each other and communicating about the women they attend to. While some women did not see the need to also include dentists in an interprofessional collaboration, others saw benefits in their incorporation as well and would consider an all-round medical team in their pregnancy as ideal.

*“I think it would be utopian to really have such a complete team*,* but I think it would really be the absolute dream and luxury for every pregnant woman. One would only benefit from it.”* (30 years, 1st child).

They outlined this with the idea that dentists could pay attention to pregnant women’s oral health and be part of the prenatal education on this topic, as they might be able to better explain the connection between oral health and pregnancy. Multiple women outlined their idea of an ideal process, where they get basic information and backgrounds by their obstetrician and midwife and a more extensive education by the dentist, as they feel those are the experts on oral health topics. Through revisiting these topics and multiple reminders by midwives, obstetricians and dentists, oral health could be better incorporated in prenatal care, as pointed out by multiple women.

*“What might make sense is if the obstetrician refers to a dental appointment among all the documents and information that come with it. And you can simply get the information from the dentist*,* as that is their area of expertise*,* I think.”* (26 years, 1st child).

#### Perspectives of experts

The interprofessional exchange was generally perceived as positive by the three interviewees as well, with the obstetrician emphasising the redundancy this can establish for pregnant women in their education on oral health.

*“I think the collaboration is good because it creates redundancy. So if the pregnant woman has heard it not only once*,* but also from the midwife*,* it reinforces the message.”* (Obstetrician).

The dentist considered the guidance on oral health in pregnancy to be interprofessional as well, stating the benefit of dentists being able to notice other issues in oral health than midwives or obstetricians could. On the other hand, the midwife noted that she might be able to mitigate fears or uncertainties that pregnant women have towards a dental visit.

*“I see great advantages*,* because there are always patients who fall through the cracks. And depending on where the patient first ends up*,* we can refer them better*,* or point out certain problems or complications. And it makes sense for everyone to be on the same page and work together towards a common goal.”* (Dentist).

The interviewed dentist also recognized inconsistency of information as an issue and empathised with pregnant women feeling irritated by different statements by multiple healthcare professionals. She proposed a joint summary of recommendations for pregnant women, pointing out the most important information for them.

*“And it makes sense that everyone is pulling together in the same direction and not the obstetrician saying one thing*,* the dentist saying another*,* and the midwife saying something different. Then of course the patients are confused and don’t know what is important and how it is meant. And it would make sense if we could formulate together what the important things are.”* (Dentist).

### Professional education

#### Perspectives of experts

The preparation to consult pregnant women on oral and dental health was different depending on the respective professional education the interviewees received. The dentist reported that pregnancy was discussed during her medical training, added however that much information was obtained through additional training.

*“It was indeed discussed that*,* for example*,* the second trimester is ideal for treatments. Things like the vena cava compression syndrome are discussed. Of course*,* which treatments are not allowed during pregnancy are also discussed. So*,* in this respect*,* quite a few things are addressed that I found to be quite relevant in practice. Everything else*,* I have to say*,* was covered through further training*,* where the topic was also included.”* (Dentist).

Neither the midwife nor the obstetrician had received information on oral and dental health during their medical training. The obstetrician reported she obtained all information through her professional association and while she does not see the necessity to implement this specific topic during medical studies, she would have appreciated an education during her specialist medical training when studying to become an obstetrician.

*“Only through the professional association and such things. Dental health wasn’t really a topic when I started with obstetrics over 20 years ago.”* (Obstetrician).

## Discussion

This study assessed women’s needs, wishes and preferences regarding the interprofessional guidance on oral health in pregnancy and aimed to identify pregnant women’s preferences regarding sources for information and consultation on this topic. Women’s perceived importance of oral health related topics was compared to the perspectives of three involved healthcare providers, a midwife, an obstetrician and a dentist. The key results of this study revealed the need for improvement of consultation on oral health in prenatal care. As stipulated by the German maternity log, oral health should be addressed by prenatal healthcare providers [11]. However, as this study shows, the execution of that does not necessarily meet the expectations of pregnant women and from their perspective could be improved in many ways. Most notably, we observed a lack of thorough information provided by the involved healthcare professionals, beyond a reminder to visit the dentist by obstetricians. Focus group participants expressed a variety of needs directed at prenatal care providers, encompassed by the general wish to be offered consultation and information on oral health in pregnancy proactively. Women wished for a personalised consultation catering to their individual knowledge and coping abilities. For information on oral and dental health, the involved healthcare providers were women’s key source, due to the general unawareness of women regarding the relevance of this topic as was previously observed by multiple studies in Germany [8, 9, 22] and other countries as well [23–25]. The lack of adequate counselling is especially concerning when considering the fact that the women in this study had a relatively high level of education and showed a strong willingness to educate themselves independently from their healthcare providers. The heightened health awareness of the study participants did not however suffice to attribute any significance to the topic of oral health in pregnancy, meaning that not necessarily health literacy but the primary prenatal care providers, midwife and obstetrician, are the essential components in the guidance on this topic. An improved guidance and more comprehensive information on oral health in pregnancy by both midwife and obstetrician could therefore emphasise its importance. Additionally, many women voiced their interest in learning about the scientific background of the connection between oral health and pregnancy as part of their prenatal care, coinciding with the positive effect an adequate health education has on health literacy, which has been demonstrated in multiple studies, showing enhanced knowledge and better implementation of beneficial health practices [24, 26].

While the interviewed obstetrician in this study recognised a primary responsibility of her profession in informing pregnant women on oral health, the ability to do so extensively is significantly limited due to time pressure in everyday practice. Midwives might be able to compensate this through their, in terms of time, more flexible approach in prenatal care [27, 28], and dentists with a more in-depth consultation on oral health. However, this might be challenging due to two aspects, one being midwives and dentists both seeing the primary responsibility with the obstetrics profession, as the interviewees stated similarly to the interviewed obstetrician herself. Secondly, this approach requires every pregnant woman to have an alternating care model, which is currently far from the reality in Germany [16], might however reinforce adequate guidance on oral health. However, in this study, the model of care did not influence the extent of women’s guidance on this topic, which might be explained by an insufficient professional education prenatal care providers receive in their respective studies regarding oral health in pregnancy [9, 13]. This results in the necessity for healthcare professionals to proactively obtain information through their professional association, which strongly depends on their personal commitment.

There was an agreement throughout focus groups as well as expert interviews regarding the importance of an early insistence to make a dental appointment by midwife and obstetrician, followed by reminders in subsequent visits. This suggests the ideal process of both midwife and obstetrician being responsible to inform women about the significance of oral health in pregnancy early on and the importance of a dental visit preferably in the second trimester, due to decreasing maternity sickness and IVCS not yet being an issue. Dentists should then present the opportunity to women for a consultation focused on their pregnancy and scientific backgrounds regarding its connection to oral health. Clear instructions on the timeline and safety of dental visits should be available to all involved healthcare providers, implemented in their respective professional education and passed on to the women in their care. A wish voiced by both focus group participants and interviewed experts alike was the implementation of a free-of-charge professional dental cleaning at least once in every pregnancy, which would give women an incentive to implement this medical service, and has been discussed by previous studies as well [23, 29]. Many German health insurance companies partly reimburse a professional dental cleaning independently from pregnancy, the results of this study show however that this is not necessarily a sufficient motivation for women to take the offer and a free-of-charge implementation in prenatal care would therefore be beneficial.

The results of this study furthermore demonstrate the overall agreement on the need for more written material providing women with information, importantly not as a substitute but in addition to a comprehensive verbal education. Written information comprised of an explanation as to the connection between oral health and pregnancy as well as the most important check-ups for mother and future child were proposed. The German children’s dental passport usually encompasses all those aspects [30] and has been positively perceived by those women in this study who received it. This result suggests the need for obstetricians to hand out the children’s dental passport, potentially as an extension to the maternity log, to all pregnant women in their care, which would guarantee the accessibility to the most important oral and dental health information for every pregnant woman.

The use of the children’s dental passport might also benefit from an integration into health insurance apps, which is also congruent with the wish of many participants for websites by governmental sources which they can trust and wish to be forwarded by their healthcare providers. The potential of utilising new media in health promotion and healthcare providers’ implementation of them in education settings has also been pointed out by other recent studies, which outlined the positive effect this can have in improving health awareness [31, 32].

The different approaches and practical settings present at the same time an opportunity for the multiple involved healthcare professionals to collaborate interprofessionally. This would open up the possibility for women to inquire about oral health-related topics in multiple settings and on different occasions, catering to their need of addressing certain topics at appropriate times when they become relevant or answer questions that have arisen over time. There was an agreement in this study between pregnant women and healthcare professionals that an interprofessional collaboration between midwife, obstetrician and dentist has the advantage of repeating and reinforcing important information. It also creates the opportunity to encounter pregnant women in different environments and the dentist can enable a consultation setting where women are able to focus on the issue of oral and dental health, without being preoccupied by the multitude of other pregnancy-related concerns. Women’s fears regarding dental visits might be mitigated by midwives or obstetricians, while dentists could take on the responsibility of a comprehensive oral health consultation and in turn, midwives could ensure the at home implementation of recommended oral hygiene practices. The ability of dental professionals to notice other oral health related issues than midwives or obstetricians was outlined by the interviewed dentist, who furthermore pointed out the advantage of interprofessional collaboration in preventing any pregnant women from slipping through the cracks in the healthcare system.

While there is a general lack of corresponding research conducted in Germany, one recent German study by Oechsle et al. questioned pregnant women on their knowledge of lifestyle-related risks, which included oral health [33]. The results of that study show a certain awareness of existing risks but a lack of knowledge regarding associated adverse effects. The authors also outline the impact of women’s socioeconomic status, concluding that women with a lower household net income had a higher risk of misjudging lifestyle-related risk factors during pregnancy [33]. Considering the importance of oral health and severity of associated APOs, a guaranteed guidance should be available to every pregnant woman, regardless of her socioeconomic status. Providing women with comprehensible, easy to navigate, evidence-based oral health information could ease the already high responsibility of being pregnant and at the same time increase the empowerment for self-decision-making and the health literacy of pregnant women. Communicating the importance of oral health in pregnancy is additionally an essential step towards a reduced risk of APOs.

While the obtained data of this study does not allow any immediate conclusion about the perspectives of pregnant women from other socioeconomic and cultural backgrounds than the study participants, it can be assumed that those who have limited health literacy might also be the ones who are affected most by the adverse effects of poor oral hygiene. Studies have shown that a lower sociodemographic as well as oral health status are associated with lower oral health literacy [34, 35]. Additionally, oral health literacy in pregnant women is associated with knowledge about oral health behaviours in young children [35, 36]. Less knowledge about offered prenatal care models might reduce access to care and limit provision of important health information, placing these women at a higher risk for APOs. This underscores the importance of improving health literacy, in order to improve oral health status in pregnant women. Future studies should therefore expand on the perspective of needs, wishes and preferences of women in prenatal care, who have lower income and educational levels, with language barriers also possibly playing a role in the quality and access to care. A recent study by Spinler et al. investigated barriers that exist for migrants in Germany towards dental treatment and prevention, and has demonstrated the need for higher cultural sensitivity in oral healthcare and the integration of migrant-specific items in the collection of health data [37]. Including a more diverse study sample could therefore be beneficial in future research assessing the needs, wishes and preferences of different population groups receiving prenatal care in Germany.

### Strengths and limitations

A bias was potentially introduced by the relatively high educational status and health literacy of study participants, who worked however in a very heterogeneous range of professional fields, which in turn is a strength of this study. A limitation might be the high number of participating women living in Northern Germany as compared to Western and Southern Germany, with no participants from Eastern Germany. Experiences in prenatal care might differ between federal states of Germany and perspectives from other states that are not covered by the place of residency of this study’s participants could not be assessed. The three interviewed healthcare experts also practiced in Northern Germany, which increased comparability with experiences of focus group participants, however, did not allow any insights into specific working conditions in other federal states. While the study sample was not diverse in terms of educational level and cultural background, it did however cover a large range in age, with women between the ages of 23 and 38. The study participants furthermore were cared for in all possible models, from exclusively obstetrics or midwifery care to an alternating care model between the two professions.

As the goal of healthcare research is encompassed by the perspectives of and benefits for its recipients, results of this study could be utilised to further develop quantitative tools, such as the Patient Benefit Index (PBI). This questionnaire reports the ratio between defined individual treatment needs and the subsequent degree of achievement of those treatment goals [38]. Such measurements could be used in reporting the benefit of interprofessional oral health counselling. Furthermore, standardised recommendations for action, specific wishes and proposed improvements to prenatal care could be directly implemented in medical and midwifery education. While the recent academisation of midwifery in Germany presents an opportunity to integrate these topics in their training, the specific wishes of women regarding the way and form of receiving information as well as the timing of consultation provide the knowledge on how to do so.

## Conclusion

At the current state of research this is the first study considering oral health in the context of interprofessional guidance in German prenatal care. The information women received on oral and dental health in pregnancy has been shown to be insufficient and the awareness regarding the health-related relevance of this issue to be lacking. Healthcare professionals are pregnant women’s key source for oral health information, which emphasises the need for an implementation of this topic in the professional education of all involved healthcare providers, especially considering the impact oral health has on pregnancy outcomes. Counselling adapted to the needs, wishes and preferences of women during pregnancy and the implementation of this topic in the education of involved healthcare professionals could contribute to improved prenatal care for pregnant women in Germany and subsequently a reduced risk of negative pregnancy outcomes as well as better long-term oral and dental health.

### Electronic supplementary material

Below is the link to the electronic supplementary material.

Supplementary Material 1

## Data Availability

The datasets used and analysed during the current study are available from the corresponding author on request.

## Abbreviations

APO: Adverse pregnancy outcome
LBW: Low birth weight
PTB: Preterm birth
LPEK: Local psychological ethics committee of the
OSF: Open science framework
UKE: University Medical Centre Hamburg-Eppendorf
SD: Standard deviation
IVCS: Inferior vena cava syndrome
PBI: Patient Benefit Index
