# Supplementary material for: Assessment of women’s needs and wishes regarding interprofessional guidance on oral health in pregnancy – a qualitative study
Source: BMC Pregnancy Childbirth. 2024 Jul 11;24:471. doi: 10.1186/s12884-024-06675-w (PMC11238511; doi:10.1186/s12884-024-06675-w)
Supplement: Supplementary file 1 — Supplementary Material 1 [file 12884_2024_6675_MOESM1_ESM.docx]

**Supplement S1:** Focus Group Guide

**Focus Group Guide**

Thank you very much for agreeing to participate in this study.

With this study we want to find out, what needs and wishes women have during pregnancy regarding the education and consultation on nutrition and oral health. For this, we want to imagine a “make-a-wish”-world together with you, meaning you can say anything that comes to mind and that you wish for, regardless of whether that service is currently offered or not.

As the education of midwives in Germany is currently changing and there is a lot of thought about possible interprofessional collaboration between different professions, the wishes you express can help to better align the care that is currently offered, with the actual needs of pregnant women.

Nutrition and oral health are important topics in pregnancy and can have an impact on how your pregnancy progresses, for example, the composition of the bacteria in your mouth may change, or you might have bleeding gums. That’s why it’s important to create an education about these relevant topics that fits the actual needs of pregnant women as optimally as possible.

To gain a comprehensive insight, we would like to look at how you have experienced education and consultation on nutrition and oral health so far, who has provided you with information and in what form, and anything else you would wish for beyond that.

We look forward to your personal opinion – there is no right or wrong. Our goal is to learn about your needs and wishes – these can be very individual. We also hope to have a pleasant conversation and a lively exchange during this session. You are welcome to comment or expand on anything another person says.

I will ask different questions and may steer the discussion in certain directions, but will try to keep my own speaking time as small as possible so that we can learn more from you.

I will record our session, which will be transcribed afterwards. This transcript will be anonymized, so that no conclusions can be drawn about your identity.

Do you have any questions?

Then I will start the recording now and we will begin with a brief introduction round.

| Main Questions | Follow-up Questions |
| --- | --- |
| Introductory Question | |
| First, I would like to get an overview and ask, what kind of care model you have in your current pregnancy? | Prenatal care shared between midwife and obstetrician or only obstetrician?  Other form of prenatal care? |
| Specific Topics: Nutrition | |
| To split our two topics today, I would like to begin with the topic of nutrition. This is not only about what you can and cannot eat during pregnancy, but also about your overall dietary habits. | |
| To what extent have you received counselling or education regarding nutrition during pregnancy and from whom?  Which topics were discussed?  If you did not receive education, what would you have wished for in that regard? | Potentially mention specific professions:   - Midwife - Obstetrician - Dietician - Family physician - Dentist |
| How did you experience this education?  What do you think about the handling of the topic of nutrition during pregnancy? | How positive/negative did you feel about the communication with you?  To what extent did you feel that your wishes were taken into account and taken seriously?  In what form were the information conveyed?  (in conversation, brochures, internet links, childbirth preparation courses) |
| To what extent did you feel that the education was sufficient?  What other resources or offerings would you have wished for? | How important is it for you to be advised on certain forms of diet?  At what point during pregnancy would you like to receive information? |
| Which nutrition-related topics or information are particularly important to you?  Which information on nutrition-related topics have been particularly helpful to you?  Where would you like to have more information? | How important is nutrition during pregnancy to you?  To what extent have you already dealt with this topic before pregnancy?  To what extent has your awareness about nutrition changed during pregnancy? |
| In times of the internet, we often obtain information through Google or social media, where it can sometimes be difficult to determine how reliable certain sources are. The sheer amount of information can also be overwhelming. | |
| How do you feel about this/experience this?  What topics do you research on the internet? | How do you deal with that? What helps you cope with the amount of information?  How do you find the information you need?  What (official) sources do you use?  What would help you filter information from the internet by relevance or credibility?  To what extent do you obtain information from your personal surroundings (family, friends)? |
| How do you make decisions regarding your nutrition during pregnancy?  To what extent does you knowledge about nutrition influence your decision on what to eat? | What is important to you when making decisions about your nutrition during pregnancy?  What influences your decision?   - Health of the baby/own health - Keeping your figure/avoiding too much weight gain - Food cravings - Gut feeling   Where do you wish for more/a different kind of support when making decisions or planning your nutrition? |
| Specific topics: Oral and dental health | |
| During pregnancy, oral health can change, for example due to hormonal changes or changes in dietary habits, which can lead to gum bleeding, for instance. Therefore, I would like to learn more about your experience with counselling and education regarding oral and dental health during your pregnancy. | |
| Introductory question: To what extent is the topic of oral and dental health during pregnancy of relevance to you? How important is this topic for you? | How do you feel about how the topic of oral and dental health is addressed during pregnancy? |
| How much consultation or education have you received on the topic of oral and dental health during your pregnancy?  What would you have wished for in this regard?  From whom did you receive information?  Did you find the education to be sufficient? | Potentially mention specific professions:   - Midwife - Obstetrician - Dietician - Family physician - Dentist   Did you bring up the topic yourself and what was the cause? |
| To what extent did you experience changes in your own oral and dental health during your pregnancy? | When would you decide to seek consultation?  Who would you prefer to receive help from?  What would contribute to relieving the decision-making process of seeking consultation? |
| Which information is particularly important to you in the education?  In what form would you like to receive this information?  How could preventive measures for oral health be integrated during pregnancy? | Information on which topics have been particularly helpful to you?  Where and on what would you wish for more information?  At what point during pregnancy would you have liked to receive education? |
| Interprofessional collaboration on both topics | |
| Finally, I would like to discuss with you how you felt overall about the care and education provided by all professional groups regarding both topics. | |
| To what extent and by whom were options for education discussed with you?  To what extent do you feel that you had sufficient time and opportunity to ask everything you wanted to discuss? | How important would it be for you to be referred to other professional groups? |
| How important would a collaboration between the different professional groups with whom you are in contact with during pregnancy be to you? | Who should be responsible for providing information on nutrition and oral health?  How important is it for you to have a clear responsibility or contact person?  In what setting would it be best for you to receive consultation on these topics?  How important is it for you to be cared for by one or more professional groups throughout the entire pregnancy? |
| To what extent do you feel that you have acquired competencies in these topics? | What in particular contributes to making you feel confident and competent?  Who has helped you with that? From whom would you like to receive help?  What would you need to feel more confident and competent? |
| At what point during pregnancy do you think an education on nutrition and oral health is the most sensible? | How have your questions and needs regarding these topics changed over time?  What information did you receive about these topics already before pregnancy?  What would help you feel well informed before and during pregnancy? |

**Supplement S2:** Developed category system reflecting the discussed topics in focus groups and interviews. Deductive: light grey background; inductive: white background.

| **Main categories** | **Subcategories** |
| --- | --- |
| Source and type of information | Experts/Health insurance app  Internet/Social media  Personal contacts |
| Oral and dental health related concerns in pregnancy | Tooth decay  Sensitivity of gums  Oral hygiene and products  Dental health of child  Scientific backgrounds  Tooth-friendly nutrition  Pregnancy-related issues  Dental treatment during pregnancy |
| Timing of consultation | |
| Prenatal oral health services in demand | Cost coverage  Offer for information/consultation |
| Lack of guidance | |
| Interprofessional collaboration | Exchange and cooperation  Consistency of information |
| Professional education of experts | |
